# Multi-omics Analysis of Primary Cell Culture Models Reveals Genetic and Epigenetic Basis of Intratumoral Phenotypic Diversity

**DOI:** 10.1016/j.gpb.2018.07.008

**Published:** 2020-03-20

**Authors:** Sixue Liu, Zuyu Yang, Guanghao Li, Chunyan Li, Yanting Luo, Qiang Gong, Xin Wu, Tao Li, Zhiqian Zhang, Baocai Xing, Xiaolan Xu, Xuemei Lu

**Affiliations:** ^1^CAS Key Laboratory of Genomics and Precision Medicine, Beijing Institute of Genomics, Chinese Academy of Sciences, Beijing 100101, China; ^2^University of Chinese Academy of Sciences, Beijing 100049, China; ^3^Invasive Pathogens Laboratory, Institute of Environmental Science and Research, Porirua 5022, Wellington, New Zealand; ^4^Department of Cell Biology, Key Laboratory of Carcinogenesis and Translational Research, Center for Molecular and Translational Medicine, Peking University Cancer Hospital and Institute, Beijing 100142, China; ^5^Department of Hepatobiliary Surgery I, Peking University Cancer Hospital and Institute, Beijing 100142, China; ^6^National Key Laboratory of Biomacromolecules, Institute of Biophysics, Chinese Academy of Sciences, Beijing 100101, China; ^7^CAS Center for Excellence in Animal Evolution and Genetics, Chinese Academy of Sciences, Kunming 650223, China

**Keywords:** Intra-individual tumor heterogeneity, Parallel primary culture, Multi-omics, Functional phenotypic diversity

## Abstract

Uncovering the functionally essential variations related to tumorigenesis and tumor progression from cancer genomics data is still challenging due to the genetic diversity among patients, and extensive inter- and intra-tumoral heterogeneity at different levels of gene expression regulation, including but not limited to the genomic, epigenomic, and transcriptional levels. To minimize the impact of germline genetic heterogeneities, in this study, we establish multiple primary cultures from the primary and recurrent tumors of a single patient with hepatocellular carcinoma (HCC). **Multi-omics** sequencing was performed for these cultures that encompass the diversity of tumor cells from the same patient. Variations in the genome sequence, epigenetic modification, and gene expression are used to infer the phylogenetic relationships of these cell cultures. We find the discrepancy among the relationships revealed by single nucleotide variations (SNVs) and transcriptional/epigenomic profiles from the cell cultures. We fail to find overlap between sample-specific mutated genes and differentially expressed genes (DEGs), suggesting that most of the heterogeneous SNVs among tumor stages or lineages of the patient are functionally insignificant. Moreover, copy number alterations (CNAs) and DNA methylation variation within gene bodies, rather than promoters, are significantly correlated with gene expression variability among these cell cultures. Pathway analysis of CNA/DNA methylation-related genes indicates that a single cell clone from the recurrent tumor exhibits distinct cellular characteristics and tumorigenicity, and such an observation is further confirmed by cellular experiments both *in vitro* and *in vivo*. Our systematic analysis reveals that CNAs and epigenomic changes, rather than SNVs, are more likely to contribute to the phenotypic diversity among subpopulations in the tumor. These findings suggest that new therapeutic strategies targeting gene dosage and epigenetic modification should be considered in personalized cancer medicine. This culture model may be applied to the further identification of plausible determinants of cancer metastasis and relapse.

## Introduction

Most large-scale cancer omics studies aim to discover functionally significant alterations that contribute to cancer phenotypes, or to characterize cancer evolution during tumorigenesis and progression before or after treatment, for potential personalized medicine [1], [2], [3]. The integration of multi-omics data and large cohorts become necessary due to the emerging cancer hallmarks based on in-depth multiple genetic and epigenetic studies on somatic tumor cells [4], [5], [6], [7]. However, it appears that when larger sample population is interrogated and massive data are produced, the number of false positive genes, which often lead to enormous complexity in interpreting molecular mechanisms, also increases remarkably [1], [8]. Therefore, although tumorigenesis is a process of phenotypic convergence, it remains challenging to identify its common drivers due to high levels of heterogeneity within and among cancer patients [2], [9].

One of the explanations is that a complex, patient-specific, genetic interplay between somatic alterations and germline background participates in tumor progression [10], [11]. For example, patients with bladder cancer that carry a germline SNP (rs2853669) have a higher survival rate when acquiring somatic mutations in the *TERT* promoter [12]. In addition, high intratumoral heterogeneity in somatic mutations leads to complicated clonal structure of tumors. Such a phenomenon has been regarded as one of plausible determinants of cancer metastasis, relapse. and treatment failure, and thus, poses challenges to personalized cancer medicine [13]. Since diversity in tumors has not been sophisticatedly considered in most drug development programs employing artificial tumor models, empirical systems that can distinguish impacts of causative intratumoral alterations from genetic background and reflect the diversity within a tumor are of essence for better prognostics and treatment.

Primary cultures of tumor cells and patient-derived tumor xenografts for cancer patients emerge as an innovative technology in preclinical tumor models and functional response assays [14], [15]. And the practice to directly characterize tumors *in vivo* and *in vitro* at multi-omics levels using patient-derived cells has been emphasized in most studies [16], [17], [18]. Due to the technical challenges in culturing cells of solid tumors, only limited number of cell clones of solid tumors can be isolated and maintained. To commendably represent the diversity and heterogeneity of tumor cell types and states (such as metastasis and drug resistance), parallel primary cultures from one or multiple tumors from a single patient are of necessity.

Two primary cultures from a primary tumor and a recurrent tumor of a patient with hepatocellular carcinoma (HCC) have been established and reported, demonstrating their clinical significance in identifying novel biomarkers and facilitating immunotherapy [19], [20], [21], [22], [23]. The high expression levels of *PBX3* and *CACNA2D1* have been validated to be associated with tumor-initiating-cell (TIC) properties in the cell clone from the recurrent tumor [22], [23]. It remains unclear whether all cells from in each of the tumors are homogeneous or possess the same characteristics, whether the phenotypic differences among the cell clones can be distinguished based on genomic alterations, and what the discriminative genomic alterations are.

In this study, we successfully established two additional cell cultures, one from primary tumor and the other from recurrent tumor. Multi-omics sequencing and cellular phenotypic characterization were performed to investigate variations in genetics, epigenetics, gene expression, cell morphology, and tumorigenicity in the four cell cultures with the same germline genetic background. We then analyzed the variations that may lead to differences in malignant behavior of tumor cells.

## Results

### Phylogeny of four cultured primary cell populations revealed by single nucleotide variations

**Figure 1:**
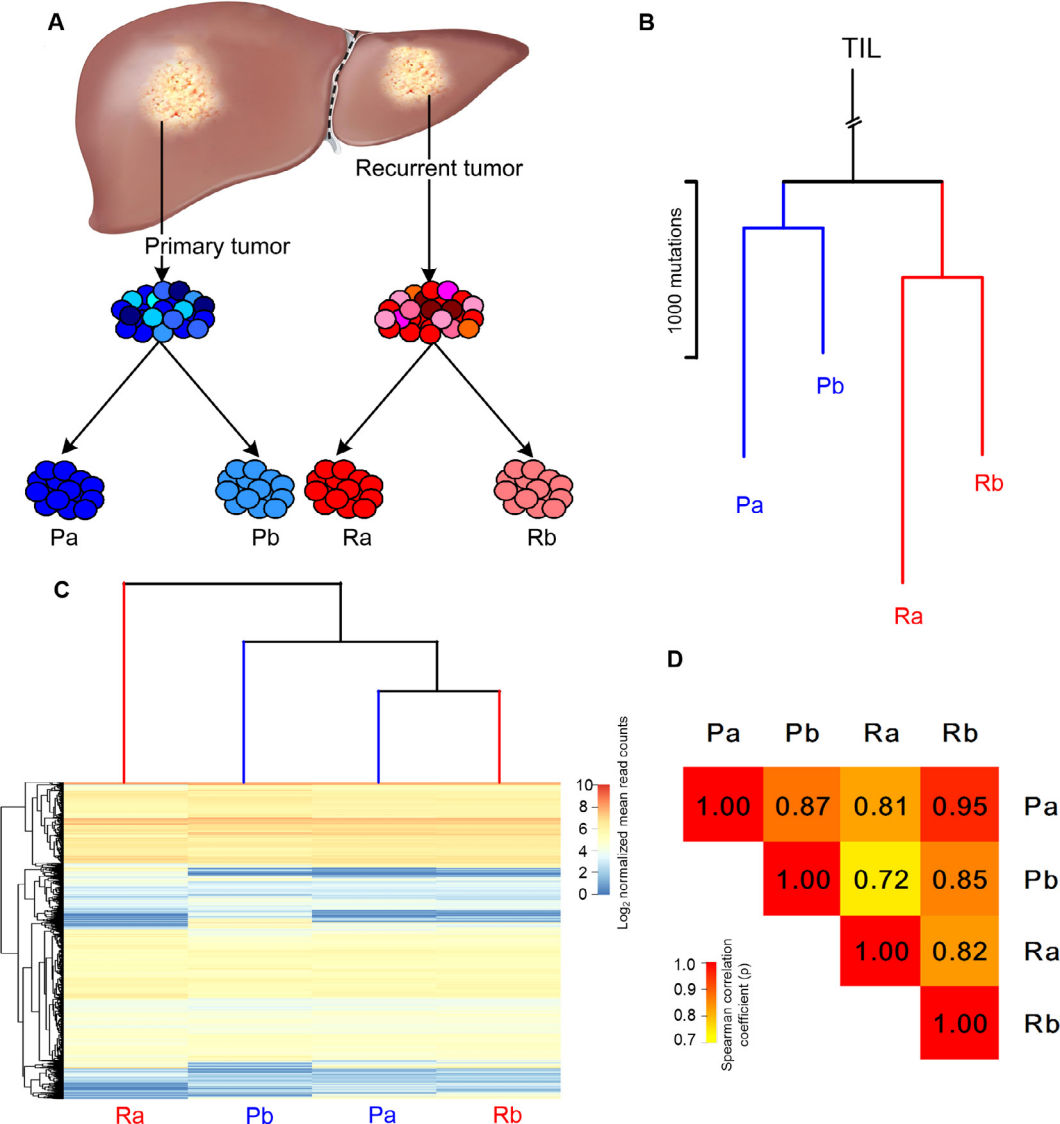
**Genetic and gene expression divergence of primary cell cultures** **A.** The four primary cell cultures from primary and recurrent tumors of an HCC patient. Primary and recurrent tumor tissues were mechanically dissociated. Primary tumor cells were initially cultured with 20% autologous serum and 10% FBS to obtain Pa and Pb, respectively. Recurrent tumor cells were initially cultured with 20% autologous serum and 10% FBS to obtain Ra and Rb, respectively. **B.** Phylogenetic tree based on SNVs calling from WGS data. The tree was anchored using a germline DNA sequence from TILs cultured from the recurrent tumor sample. Blue and red lines represent primary and recurrent tumors, respectively. The branch length from the (TILs) to the common ancestor is not shown to scale because of the great distance. The SNVs located in LOH regions were excluded in this analysis. **C.** Divergence of transcriptional profiles of cell cultures. Hierarchical clustering of the four cell cultures was performed based on normalized mean read counts (log_2_-transformed) for genes that were expressed (read count > 1) in at least one sample. The heatmap depicts the expression of genes on the log_2_ scale (rows) from each sample (columns). **D.** Heatmap of the Spearman correlation coefficients (ρ) of transcriptomes between any two of the four samples. Genes expressed (read count > 1) in at least one sample were used. HCC, hepatocellular carcinoma; FBS, fetal bovine serum; SNV, single nucleotide variation; WGS, whole-genome sequencing; TIL, tumor-infiltrating lymphocyte; LOH, loss of heterozygosity.

Primary cell cultures from primary (Pa) and recurrent (Ra) tumors of an HCC patient have been described previously [19], [20], [21], [22], [23]. To further characterize the potential heterogeneity and clonal diversity between cell populations from these tumors, two replicates, one from primary tumor (Pb) and the other from the recurrent tumor (Rb), were successfully obtained in the present study (Figure 1A, see Materials and methods). To examine the genetic divergence among these cell cultures, Pa, Pb, Ra, Rb and tumor-infiltrating lymphocytes (TILs, from recurrent tumor) were subjected to whole-genome sequencing (WGS), reaching > 60 × coverage, and whole-exome sequencing (WES) was also performed for Pa, Pb, Ra, and Rb, reaching > 100 × coverage. The TILs were used as a matched normal control of this patient for somatic variation calling from both WGS and WES data.

**Supplementary Figure S1:**
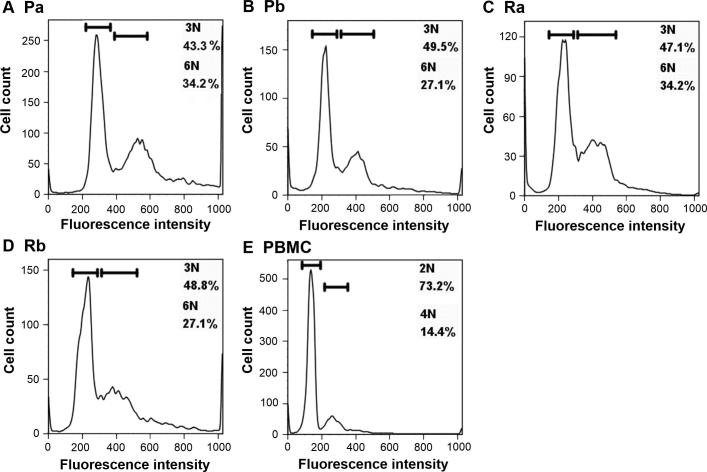
**Ploidy analysis for cell clones using flow cytometry** Pa cells. B. Pb cells. C. Ra cells. D. Rb cells. E. Human PBMC as normal cell control. PBMC, peripheral blood mononuclear cell.

**Supplementary Figure S2:**
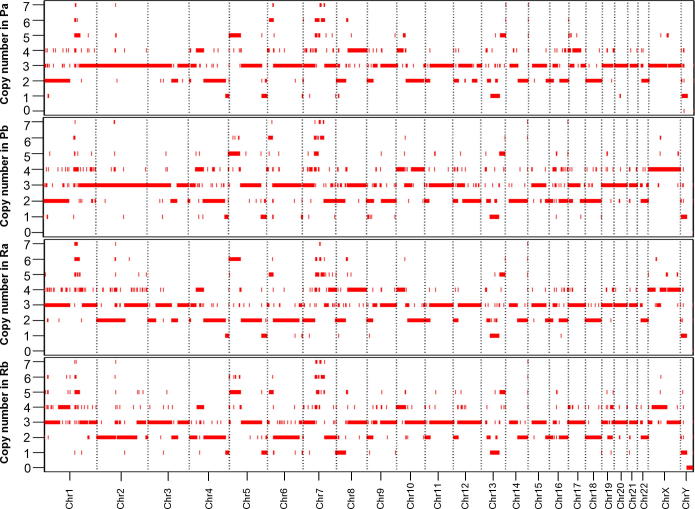
**Copy number profiles showing the genetic variations in the four cell cultures**

Genome ploidy, copy number alterations (CNAs), and losses of heterozygosity (LOHs) were detected based on the WGS data (see Materials and methods). The ploidy of these cells was estimated as triploid and further validated using flow cytometry DNA ploidy analysis (Figure S1), which is consistent with previous observations based on karyotyping of Pa and Ra [20]. The CNA profiles revealed a high level of genetic divergence among the four cell cultures. Almost all chromosomes were subject to copy number gain or loss in these cells (Figure S2). Among all 14,813 CNAs and LOHs, 22 variations (0.15%) on chromosomes 1, 3, 4, 7, 9, 10, 14, 16, 17, and 18 were shared (with the same breakpoint and the same absolute copy number) among all cell cultures (Table S1).

**Supplementary Figure S3:**
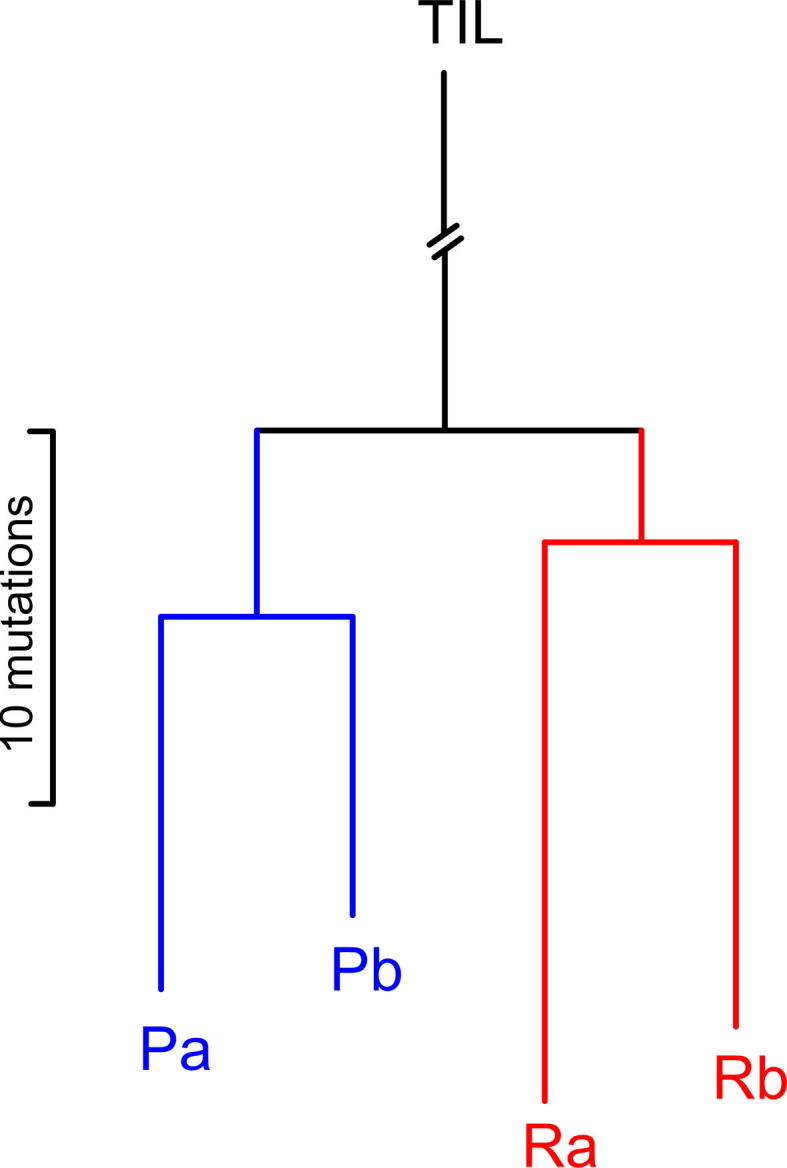
**Phylogenetic tree based on SNVs called from WES data** The tree was anchored using a germline DNA sequence from TILs (cultured from the recurrent tumor sample). Blue and red lines represent primary and recurrent tumors, respectively. The branch length from the TILs to the common ancestor is not shown to scale because of the great distance. SNVs located in LOH regions were excluded in this analysis.

We performed single nucleotide variation (SNV) calling in both of the WES and WGS data from the four cell cultures (see Materials and methods). Among the total 41,484 SNVs that were identified from WGS data, 30,937 SNVs were commonly shared and 10,545 SNVs were polymorphic (Table S2). Based on the validation data from Sequenom genotyping, we identified 341 commonly shared SNVs and 103 polymorphic variations in WES data (Table S3, see Materials and methods). About 94.6% SNVs that were identified in WES data can be detected in WGS data. The presence of shared CNAs and SNVs indicates that these four cell cultures represent four subclones originating from a common ancestor. Considering that LOH events may result in loss of mutant alleles, we further used the SNVs located out of LOH regions to construct a phylogenetic tree. Based on the WGS data, the phylogenetic tree of Pa, Pb, Ra, and Rb was consistent with the origin of the primary cell cultures. Two groups derived from a common ancestor were observed. One group included Pa and Pb, which were cultured from the primary tumor, whereas the other included Ra and Rb from the recurrent tumor (Figure 1B). Eleven known driver genes in HCCs, *RPS6KA3*, *NTRK3*, *TERT*, *TSC2*, *ARID1A*, *KMT2D*, *KMT2B*, *KMT2C*, *IL6ST*, *NFE2L2*, and *EPHA4* [24] were identified in the trunk of the tree, whereas only two genes, *NFE2L2* and *EPHA4*, were detected on the branches shared by Pa and Pb. These data indicate that in this case, mutations in functionally significant genes in HCC might have contributed to the progression of carcinogenesis in the common ancestor of these cell populations rather than account for their phenotypic differences. Additionally, the phylogeny based on the WES data showed the same pattern as the WGS data (Figure S3).

### Discrepancy between the phylogenetic tree and the divergence revealed by the transcriptional profiles

Previous studies have shown that the Ra cells from the recurrent tumor were largely tumor-initiating cells (TICs), while the Pa cells from the primary tumor were not [19], [22], [23]. Since both Ra and Rb were derived from the recurrent tumor after the patient underwent combination chemotherapy [19], they were supposed to be more aggressive and malignant than Pa and Pb due to the treatment selection pressure, as suggested by the previous studies [25]. It could be inferable that cellular characteristics might be similar in Ra and Rb, which were genetically clustered together, and might be distinguished from that of Pa and Pb at the transcriptional level.

RNA-seq was performed in the four cell cultures with two biological replicates for each (Pearson correlation coefficients are ∼0.95 between the replicates of each sample), followed by the detection of differentially expressed genes (DEGs) among the samples (see Materials and methods; Table S4). Notably, we observed that the divergence of the transcriptional profiles in the four cell populations exhibited significant disparity from the genetic phylogeny (Figure 1C, see Materials and methods). Pa was closely clustered with Rb, rather than with Pb, whereas Ra diverged markedly from the other three cell cultures. The Spearman correlation coefficient (ρ) between Ra and Pb (ρ = 0.72) was the lowest in the comparisons between the four cultures (Figure 1D).

**Supplementary Figure S4:**
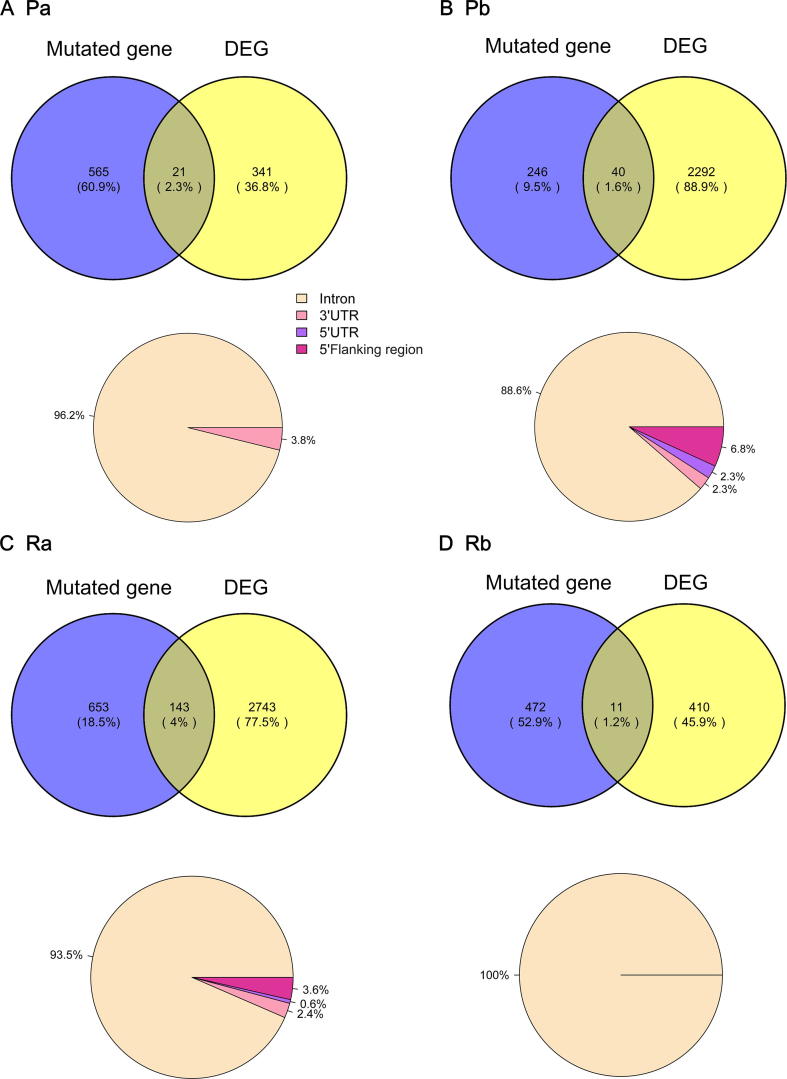
**Overlap between sample-specific mutated genes and DEGs for each sample** Overlap between sample-specific mutated genes and DEGs for Pa (A), Pb (B), Ra (C), and Rb (D). *P* values were derived from binomial test. The percentage of overlapping genes is significantly lower than the expectation (*P* < 2.2E–16 for all groups). The pie charts depict the locations of the gene mutations that overlapped with the DEGs in each sample.

Furthermore, we tested whether genes with sample-specific mutations showed significant changes in gene expression compared with other samples. Among the overlap between sample-specific mutated genes and DEGs, mutations were detected in non-coding regions, including introns, untranslated regions (UTRs), and flanking regions. Mutations in these regions can influence transcription, splicing, or stability of mRNAs [26], [27], [28], [29], [30]. Therefore, our results suggest that sample-specific gene expression might result from the mutations in the corresponding non-coding elements. However, there is no evidence showing significant overlaps between sample-specific mutated genes and DEGs for all the samples (Binomial test, *P* < 2.2E−16, Figure S4), suggesting that most of the heterogeneous SNVs among the lineages within the patient are functionally insignificant, which is consistent with previous studies [31], [32], [33].

### Consistency between the divergence revealed by DNA methylation and transcriptional profiles

Heterogeneity of DNA methylation can reflect tumor clonal evolution, and a high correlation coefficient has been observed between genetic and epigenetic distance matrices for all specimens within tumors [34], [35]. Additionally, DNA methylation plays a critical role in the regulation of gene expression, and its variation is correlated with corresponding gene expression variations across compared samples [36]. To examine the discrepancy between the lineage relationship and the transcriptional divergence of the cell cultures, we investigated the divergence pattern of DNA methylation. Based on the transcriptional profiles, differentially originating Pa and Rb were closely clustered; meanwhile, both Pb and Ra were separated from their respective same-origin clones. Hence, besides Pa and Rb, either Ra or Pb was required to interpret the inconsistence between phylogenetic tree and transcriptional divergence. Compared to Pb, Ra was more divergent from Pa and Rb at expression level. Therefore, we performed whole genome bisulfite sequencing (WGBS) for Pa, Ra, and Rb, which exhibited discrepancy between phylogenetic relationship and transcriptional pattern. That is, Ra and Rb clustered together in the phylogenetic tree but the hierarchical clustering of their transcriptional profiles was separated; in contrast, Pa and Rb were separated in the phylogenetic analysis but clustered together in the transcriptomic analysis.

**Figure 2:**
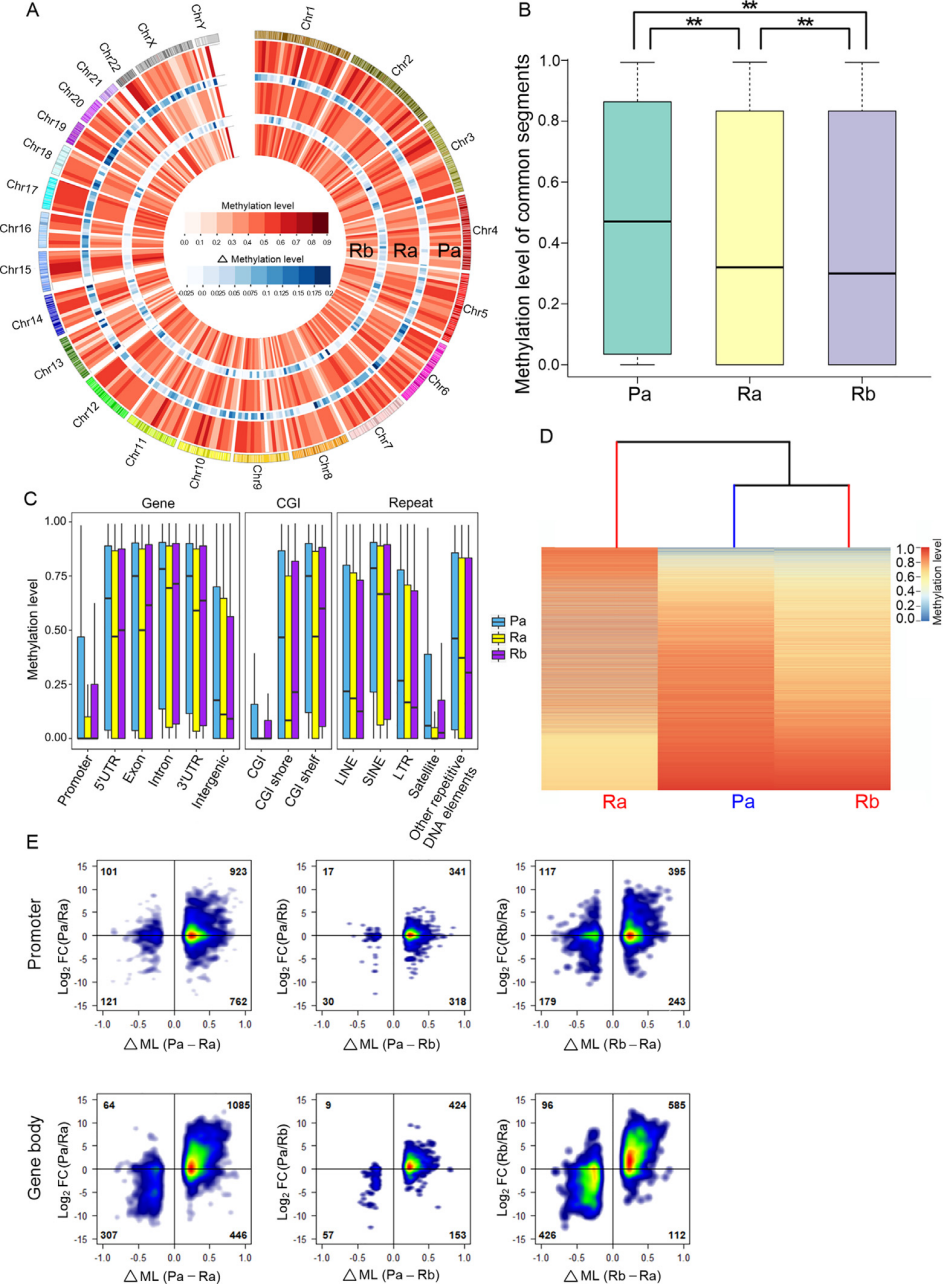
**Characterization of DNA methylation in Pa, Ra, and Rb** **A.** Circos representation of the whole-genome DNA methylation levels of the cell cultures. The outermost circle represents the chromosome locations. Different shades of red represent methylation levels averaged in 10-Mb genomic windows, and different shades of blue represent the difference in methylation levels between inner and outer layers. Inner heatmaps indicate changes in DNA methylation shown in color gradients. A gradual loss of methylation compared with that in Pa is observed (from outer to inner, Pa, Ra, and Rb). **B.** Boxplot of common CpG site methylation levels in cell cultures. ** indicates highly significant differences between any two samples (*P* < 2.2E − 16, Wilcoxon test). **C.** Boxplot of DNA methylation levels for multiple functional genomic categories in cell cultures. **D.** Divergence of the methylomes of the cell cultures. Hierarchical clustering was based on the methylation levels of differentially methylated CpG sites (≥ 10 ×) among samples. The heatmap depicts the CpG site methylation level (rows) in each sample (columns). **E.** Density plot of DMPs/DMGBs with the changes in the expression of associated genes between the compared samples. The horizontal and vertical axes represent differences in methylation levels (Δ ML) and expression changes (FC), respectively. Density is color-coded; red indicates a higher density and blue indicates a lower density. CGI, CpG island; LINE, long interspersed nuclear element; SINE, short interspersed nuclear element; LTR, long terminal repeat; FC, fold change; DMP, differentially methylated promoter; DMGB, differentially methylated gene body .

Analysis of genome-wide CpG methylation showed that the average methylation levels for Pa, Ra, and Rb were 0.460, 0.409, and 0.407, respectively, decreasing from primary tumor derived Pa to recurrent tumor derived Ra and Rb (Figure 2A). This observation is consistent with a previous study showing that global DNA hypomethylation occurred during the progression of HCC [37]. Significant differences in the extent of DNA methylation were observed between each pair of samples (pair-wise Wilcoxon test, *P* < 2.2E−16, Figure 2B), indicating heterogeneous modification of DNA methylation in these cell cultures.

We subsequently subdivided the whole-genome methylation events into multiple functional genomic categories to determine whether Pa, Ra, and Rb exhibited different methylation levels in these categories (see Materials and methods). As shown in Figuere 2C, Pa was significantly hypermethylated in all categories, whereas Ra was significantly hypomethylated in potential regulatory regions including promoter, gene body (containing 5′UTR, 3′UTR, exon, and intron), as well as CpG island (CGI) regions, and Rb was significantly hypomethylated in the intergenic and some repeat regions (Table S5, pair-wise Wilcoxon test, *P* < 1.0E−14 for all 3 samples).

**Supplementary Figure S5:**
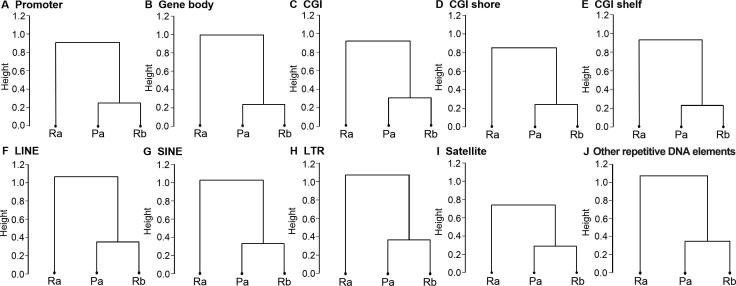
**Patterns of methylome divergence for multiple functional genomic categories** Methylome divergence pattern in promoter (A), gene body (B), CGI (C), CGI shore (D), CGI shelf (E), LINE (F), SINE (G), LTR (H), satellite (I), and other repetitive DNA elements (J). The methylation levels of DMFs were used for hierarchical clustering of samples. Height denotes the dissimilarity among samples.

Hierarchical clustering of the methylomes of these cell cultures (see Materials and methods) revealed that Rb and Pa were closely clustered, while Ra was isolated, which was consistent with the transcriptional clustering pattern but inconsistent with the phylogenetic relationship (Figure 2D). We further analyzed the DNA methylation patterns in different genomic categories. Accordingly, in all categories, Ra displayed a unique methylation profile, differing from that of Pa or Rb, as confirmed by the hierarchical clustering of significant differentially methylated functional genomic categories (DMFs; see Materials and methods; Figure S5).

### Correlation between the variations in copy number, DNA methylation, gene expression

**Supplementary Figure S6:**
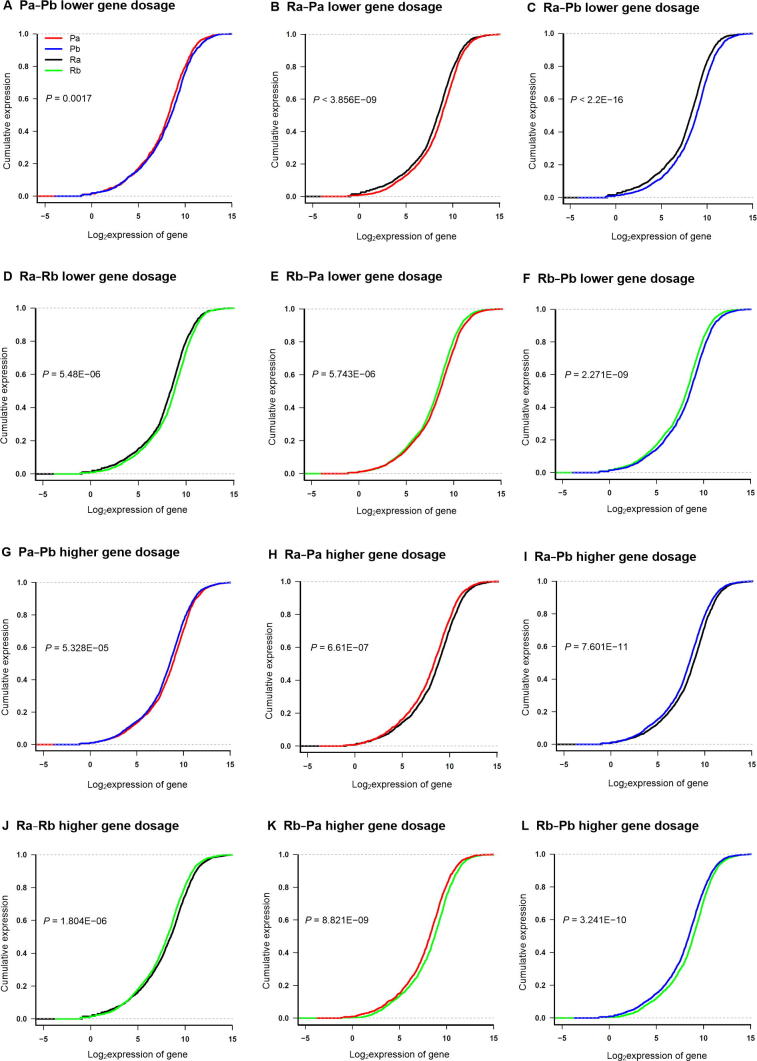
**Cumulative distribution of gene expression for genes in regions with CNAs between any two samples** The X-axis denotes the gene expression level (read counts) on the log2 scale, and the Y-axis denotes the cumulative proportion of gene expression. The P values were derived from the one-sided Kolmogorov−Smirnov test. Genes showing absolute copy number differentiation between the compared samples were used to plot the cumulative distributions. A.−F. Cumulative distribution of the expression of genes located in regions of copy number loss for pair-wise comparisons, Pa vs. Pb, Ra vs. Pa, Ra vs. Pb, Ra vs. Rb, Rb vs. Pa, and Rb vs. Pb. G.−L. Cumulative distribution of the expression of genes located in regions of copy number gains for pair-wise comparisons, Pa vs. Pb, Ra vs. Pa, Ra vs. Pb, Ra vs. Rb, Rb vs. Pa, and Rb vs. Pb.

CNAs can affect gene dosage by altering the number of gene copies in the genome. Consistency between the changes in the mRNA expression and gene copy number has been reported in multiple types of cancers [38], [39], [40]. Herein, we investigated the correlation between gene copy numbers and their expression levels in the four cultures (see Materials and methods). We observed significant consistency between the copy numbers and expression levels of these CNA genes. Genes located in regions of copy number gain and loss showed significant increase and reduction in expression level (Kolmogorov-Smirnov one-tailed test, *P* < 0.05, Figure S6), respectively, suggesting that CNAs partially contribute to the divergence in gene expression between the primary cell cultures.

Promoter and gene body methylations are negatively and positively associated with gene expression levels, respectively [41], [42], and the observed methylation patterns are consistent with transcriptional divergence. To further investigate whether differential methylation in these regions was involved in regulating gene expression in these cell cultures, we correlated the differences in methylation (ΔML) at differentially methylated promoters/gene bodies (DMPs/DMGBs) with the changes in the expression of associated genes (presented as fold change) between the samples.

In all comparisons, DMPs and DMGBs showed different density distributions. The DMP genes were predominantly distributed around the X-axis in the right-hand quadrants, indicating that their expression level was not altered according to promoter methylation variations, whereas the DMGB genes were predominantly distributed in quadrant 1, implicitly demonstrating a positive correlation between expression changes and alterations in gene body methylation (Figure 2E). The Pearson correlation coefficients between the differences in DMGB methylation (ΔML) and alterations in associated gene expression (fold change) in Pa *vs.* Ra, Pa *vs.* Rb, and Rb *vs.* Ra were 0.608, 0.502, and 0.713, respectively, which are highly positive and significant (*P =* 2.26E−192, 2.37E−42, and 5.95E−190). These results are consistent with the observation that gene body methylation is associated with active gene transcription [42], [43]. Our results also indicate that gene body methylation was more closely associated with gene expression than promoter methylation, which is consistent with the idea that the “gene body methylation is a stronger indicator of expression class than promoter methylation”, as described for human samples and cell lines [41]. In prostate cancers, gene expression was not obviously associated with promoter methylation levels between tumor samples within individuals [44].

### CNAs and variations in DNA methylation jointly confer the phenotypic differences among the cell cultures

The transcriptional profile of Ra differed markedly from those of Pa, Pb, and Rb, indicating that Ra has a unique functional phenotype. We subsequently identified 2886 Ra-specific DEGs (see Materials and methods; Table S4). According to Gene Ontology (GO) analysis, these DEGs are over-represented in 202 biological processes (Table S6). Among the top 10 significant terms, most terms are highly associated with tumor development and progression, including angiogenesis, extracellular matrix organization, positive regulation of cell migration, positive regulation of GTPase activity, substrate adhesion-dependent cell spreading, MAPK cascade, and regulation of apoptotic process [45]. Consistent with previous studies, up-regulated genes identified in Ra include the oval cell marker *AFP* [19], [20], the metabolic marker *ABCG2* [19], the embryonic cell lineage markers *FOXA2*, *IPF1*, and *ISL1*, the stem cell-associated gene *PARD6A* [19], the newly identified functional liver TIC marker *CACNA2D1* [22], and the TIC driver gene *PBX3* [23]. In particular, the last two genes are demonstrated to be highly associated with TIC properties of tumor cells in HCC [22], indicating that Ra is endowed with stronger tumorigenic capabilities than the other three cell populations, including the recurrent tumor-derived Rb.

Our previous analysis has shown that CNAs and DNA methylation changes are potentially associated with the corresponding gene expression variations observed in these cultured primary cell populations, suggesting their roles in affecting the transcriptional profiles of these cell populations. To detect the contribution of CNAs and gene body methylation to the Ra-specific functional phenotype, among 330 Ra-specific CNA genes overlapping with DEGs, we identified 249 genes (75.45%) showing consistency in CNAs with gene expression presenting the same directional change as CNA-driven genes. Among the 580 Ra-specific DMGB genes overlapping with DEGs, we identified 540 genes (93.10%) showing consistency in gene body methylation with gene expression presenting the same directional change as DNA methylation-driven genes, including 25 genes overlapping with CNA-driven genes (Table S7).

**Figure 3:**
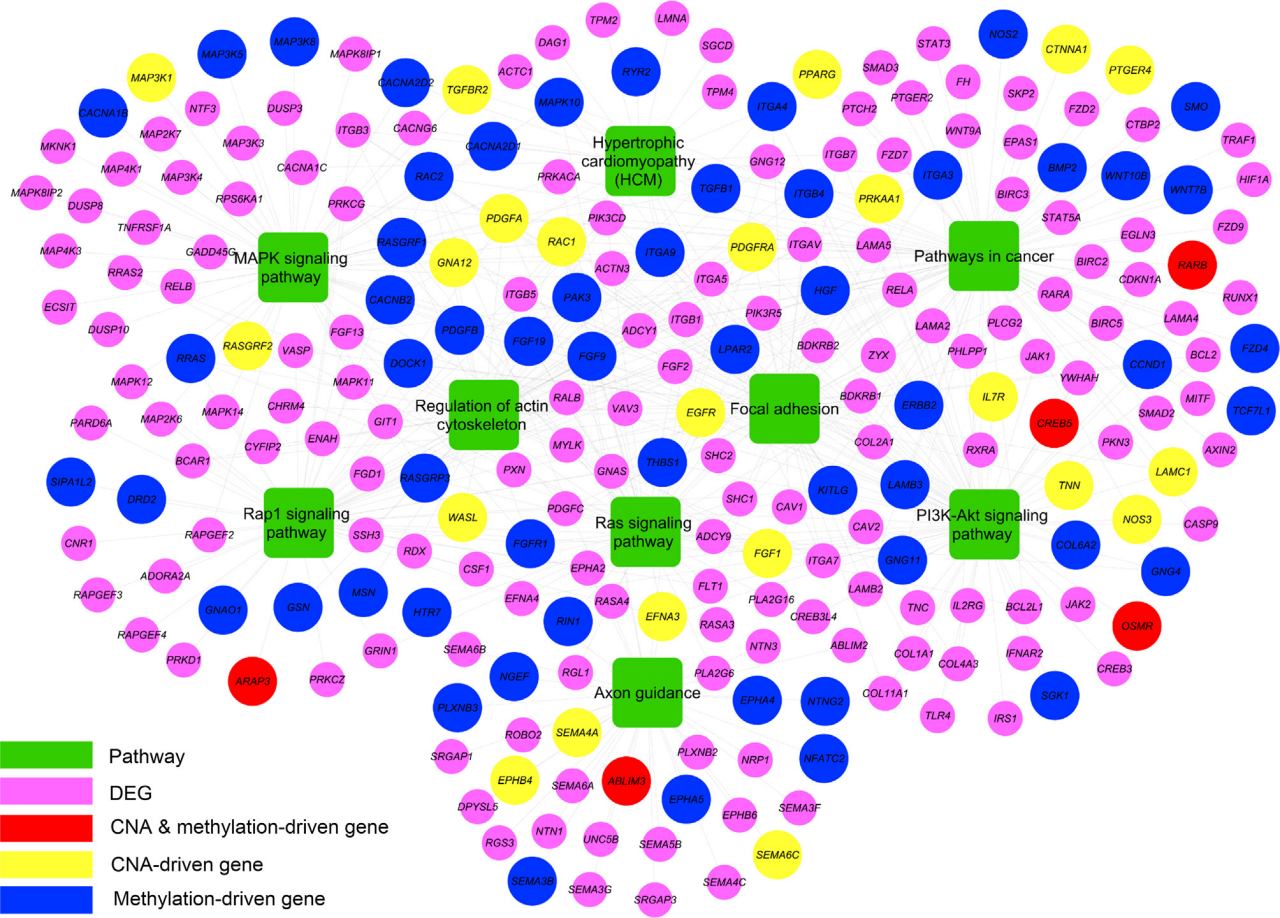
**Characterization of the roles of CNAs and variations in gene body DNA methylation in cell cultures** Significantly enriched pathways of CNA/DNA methylation-driven genes. The genes used to construct the network included CNA/DNA methylation-driven genes and other Ra-specific DEGs that also participate in these pathways. CNA, copy number alteration; DEG, differentially expressed gene.

Pathway enrichment analysis revealed the significantly enriched KEGG pathways among all the 764 CNA/DNA methylation-driven genes, as shown in Figure 3 and Table S8 (see Materials and methods), indicating the significant biological roles of CNAs and differential gene body methylation in exerting direct and indirect effects on gene expression in these cell populations. Notably, these pathways can be divided into two groups. Group 1, including the pathways in cancer, MAPK signaling pathway, PI3K-Akt signaling pathway, and Ras signaling pathway [46], [47], [48], is involved in cell cycle progression, cell proliferation, apoptosis, and tumorigenesis. Group 2, including the Rap1 signaling pathway, focal adhesion, regulation of actin cytoskeleton, and axon guidance, is involved in cytoskeleton organization, cell adhesion, regulation of cell morphology, as well as cellular assembly and organization [49], [50], [51], [52]. These two groups of pathways, which are largely affected by CNAs and gene body DNA methylation changes, are associated with cell morphology and tumor-initiating properties, suggesting that CNAs and gene body DNA methylation might jointly confer the distinct Ra phenotype. The TIC marker, *CACNA2D1* [22], a DNA methylation-driven overexpressed gene in Ra, further highlights the functional role of gene body methylation in the formation of Ra phenotype.

### Characterization of cell morphology and tumorigenicity in Ra cells

**Figure 4:**
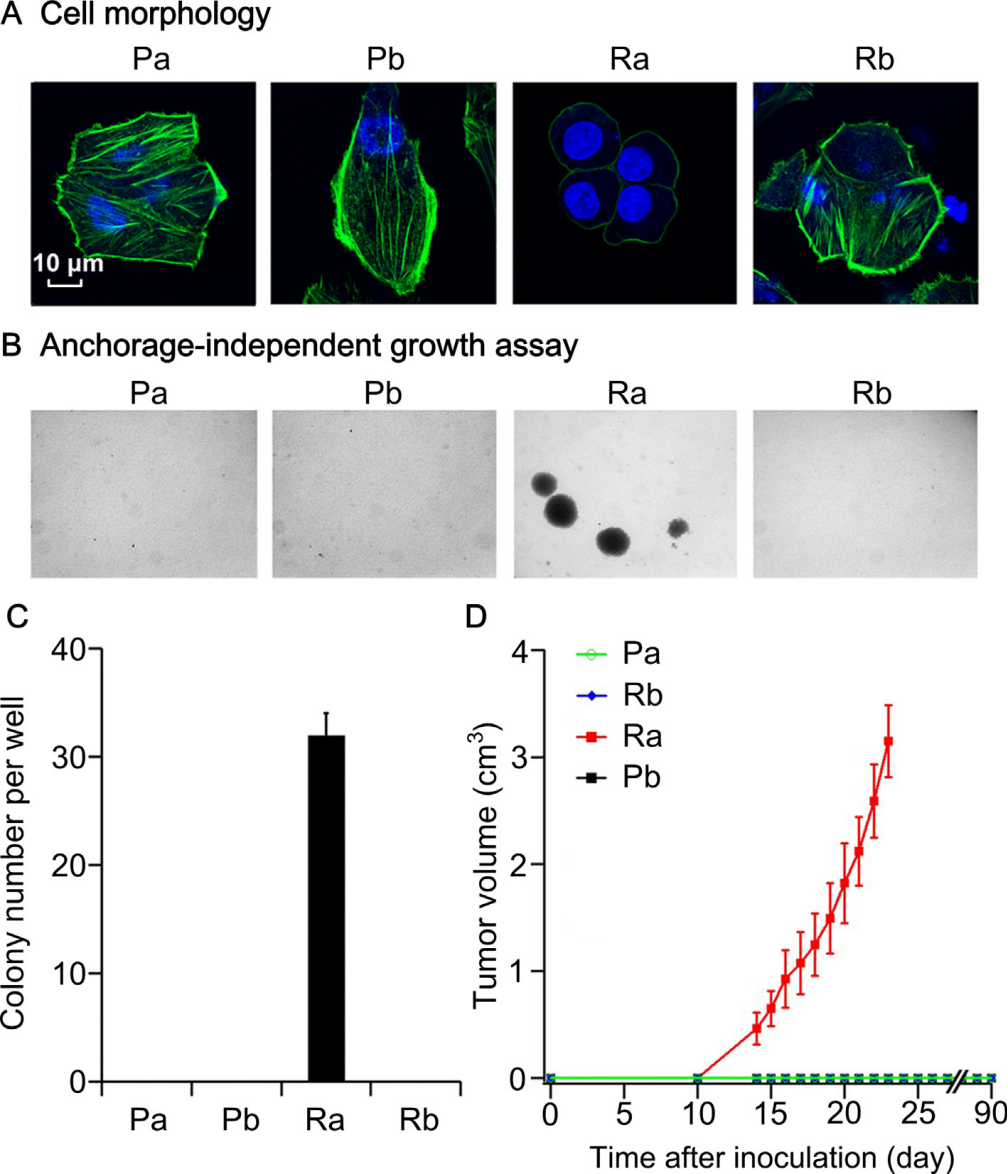
**Specific cell morphology and tumorigenicity of Ra compared with Pa, Pb, and Rb** **A.** Morphology of Pa, Pb, Ra, and Rb. Green indicates phalloidin-stained F-actin, and blue indicates the cell nucleus stained with DAPI. Bar = 10 μm. **B.** Soft agar assays (40 ×). Colonies were microscopically visualized. **C.** Quantification of colonies formed in each well. 3 wells per group (repeated twice). Data are shown as mean ± SEM. **D.** Growth curves of tumors after subcutaneous injection of Pa, Pb, Ra, and Rb cells into the armpits of each NOD/SCID male or female mouse (4–6 weeks old). Tumor formation was monitored weekly after transplantation. Two weeks later, tumors were detected only in the Ra-transplanted mice. Ra tumor was monitored for the continuous growth and was removed on day 25 due to the large tumor size. No tumors were formed in the mice transplanted with Pa, Pb, or Rb, even after 90 days. Values are presented as mean ± SEM (5 mice in each group).

We further determined whether Ra presented a distinct phenotype in terms of cell morphology and tumorigenic capability among these four cell populations. We first analyzed the morphology of these primary cells (see Materials and methods). Pa, Pb, and Rb cells grew adherently, displaying a typical epithelial morphology with polygonal shapes. In contrast, Ra cells were round in shape, adhered loosely to the flask, and formed spheres during passaging. To clearly show that the cytoskeleton maintains cell morphology and cell adhesion, we stained the cells with phalloidin to visualize a major cytoskeletal element. As expected, enriched F-actin filaments were distributed in the cytoplasm and under the cell membranes in Pa, Pb, and Rb cells; however, only a few F-actin filaments were detected under the cell membranes of Ra cells (Figure 4A). This unique morphology might be associated with the malignant behavior of Ra, as malignant transformation is frequently characterized by alterations of the cellular cytoskeleton, which results in the deficiency in cell adhesion [53]. The observation of DAPI-stained nuclei showed that Ra cells presented the highest nuclear-cytoplasmic ratio (Figure 4A). An increased nuclear-cytoplasmic ratio is typically observed in various types of human carcinomas, including hepatocarcinoma cells [54], which additionally supports a higher degree of malignancy in Ra cells.

Furthermore, we sought to characterize the clonogenicity of these four cell populations in the soft agar (see Materials and methods). Ra cells were able to grow as spheres in soft agar, while others failed (Figure 4B and C). Next, 5 × 10^6^ cells from these four primary cultures were transplanted subcutaneously into NOD/SCID mice to test their tumorigenicity (see Materials and methods). Ra cells were able to initiate tumor formation, whereas under the same condition, no tumorigenicity was detected in the other three samples (Figure 4D), which is consistent with the up-regulation of TIC-associated markers in Ra, *i.e.*, *CACNA2D1* and *PBX3* [22], [23]. Our data also revealed up-regulation of the putative hepatic stem/progenitor cell marker, *CD56*, in Pb. The non-tumorigenicity of Pb suggests that unlike *CACNA2D1*, *CD56* was not associated with TIC properties of tumor cells in HCC, consistent with our previous study showing that the marker *CD56* was not enriched in TIC populations [19]. The Ra cells from the recurrent tumor showed distinct cellular phenotypes, which is consistent with the prediction based on CNAs and gene body differential methylation, suggesting that CNAs and gene body DNA methylation might play vital roles in these biological properties by directly or indirectly regulating the expression of the corresponding genes.

## Discussion

The accumulation of somatic aberrations and the dynamics of subclonal changes over time shape the evolutionary process of tumors and confer tumor heterogeneity. Since high level of heterogeneity within and between patients is a major obstacle to successful cancer therapy [13], it should be taken into account when performing in-depth exploration of tumors or designing treatment strategies. Patient-derived primary tumor cells that can provide high-fidelity data derived from specific patient, have the potential to translate *in vitro* findings to *in vivo* models and ultimately to personalized therapy [14]. However, obtaining multiple primary cell clones to understand tumor heterogeneity remains challenging, due to the technical specialties in the primary cell culture. This study is the first to compare genomic and phenotypic differences among multiple primary cell cultures derived from primary and recurrent tumors from the same patient using multi-omics analysis.

The four cell cultures originated from a single ancestor, but presented heterogeneous, whereas the clustering relations generated via genomic, transcriptional, and epigenomic variations were discrepant. Phylogenetic relationships based on SNVs accurately reflected the clonal origin, consistent with previous studies showing that genetic variations can be used to reconstruct clonal evolutionary relationships among different tumors [55], [56]. We also observed that the phylogenetic tree based on SNVs identified in the WGS data is consistent with the pattern based on the WES data (Figure 1B and Figure S3), indicating that WES data is enough for reconstructing the authentic phylogeny. Transcriptional divergence that indicates phenotypic variation was inconsistent with the phylogeny. Significant overlap between sample-specific mutated genes and DEGs for each sample was not observed (binomial test, Figure S4), supporting previous report that most of the intratumoral heterogeneous SNVs appear to be functionally insignificant [31], [32], [33]. Inconsistency between genetic phylogeny and the transcriptional clustering pattern has also been observed in previous studies in glioblastoma (GBM) [57], [58] and melanoma [59]. The expression clustering patterns of both single cells and single-cell-derived clones of GBM did not show clustering according to tumor origin [57], [58]. Harbst et al observed that two evolutionarily similar regions displayed different gene expression subtypes in melanoma [59].

Further analysis revealed that CNAs and changes in gene body DNA methylation modifications were associated with the expression divergence of these cell populations. An alternative explanation for this observation is that CNAs can affect gene dosage by altering the number of gene copies in the genome, thereby conferring a stronger impact on gene expression changes than point mutations [60]. Additionally, DNA methylation plays critical roles in the regulation of gene expression, and gene body methylation has been associated with active gene transcription [42], [43]. In addition to the direct effect on the expression of the corresponding genes, CNAs and gene body DNA methylation changes might indirectly affect genes through participating in certain pathways and, thus, jointly confer distinct cellular phenotypes to Ra.

Analysis of the whole genome showed that the recurrent tumor-derived Ra and Rb cells presented significant hypomethylation compared to primary tumor-derived Pa. Significant overexpression of *TDG* leads to the demethylation of a methylated construct transfected in cultured (HEK) cells [61]. Up-regulated *TDG* may contribute to low methylation levels in Ra and Rb than in Pa. In the present study, we observed that Rb was closer to Pa and was largely separated from Ra based on genome-wide DNA methylation patterns. Ra displayed a unique methylation profile, differing from those of Pa and Rb in all functional genomic categories. These results suggest that Ra has undergone the DNA methylation changes required for progression to higher malignancy. For example, demethylation of potential regulatory regions, including gene bodies, might account for the distinct transcriptional profile of Ra.

Despite the fact that Ra and Rb cells were both derived from the recurrent tumor of the same patient, Rb did not present TIC properties like Ra, suggesting that functionally heterogeneous subclones exhibit in the recurrent tumor following the treatment. Although intratumoral heterogeneity was shown to be reduced upon therapy [62], [63], there is still a need to detect multiple clones within recurrent tumors. However, further studies are needed to determine whether the principles discovered here apply to other patients. From systematic analysis of one patient-derived primary culture, we observed the cellular functional heterogeneity is more closely associated with CNAs and differential gene body methylation than subclonal SNVs, which, if validated in more individuals, will guide the development of therapeutic strategies targeting not only SNVs but also CNAs and variations in DNA methylation. These primary cultures may be applied to further identifying the plausible determinants of cancer metastasis, relapse, and treatment failure. The systematic strategy, combining multi-omics data with the measurement of cellular phenotypes in parallel primary cell cultures from the same patient, could be applied to identifying malignant subpopulations and characterizing variations related to malignancy, and further extended to the field of personalized cancer therapy.

## Materials and methods

### Patient information

Information of the HCC patient previously described [19], [20]. Briefly, a 47-year-old male with chronic hepatitis B virus (HBV) infection initially underwent HCC resection of the right lobe of liver, followed by treatment with combination chemotherapy for two transarterial chemoembolization procedures. The patient underwent left lateral lobectomy 7 months later, due to HCC recurrence on the left lobe of liver. Both primary and recurrent tumor tissues were obtained from this patient for research proposes at Peking University Cancer Hospital. This study was approved by the Human Research Ethics Committee of Beijing Institute of Genomics, Chinese Academy of Sciences (CAS), and Peking University Cancer Hospital. Informed consent was obtained from this patient as previously described [19], [20].

### Primary culture of tumor cells and TILs

We established two primary cell cultures from the above HCC patient, designated Hep-11 (from the primary tumor) and Hep-12 (from the recurrent tumor), as previously described [19], [20], and these cells were renamed as Pa and Ra in the present study. Briefly, both primary and recurrent tumor specimens were dissociated and layered onto a 75%/100% two-step Ficoll gradient. After centrifugation, some of these mechanically dissociated tumor cells enriched in the upper interface and lymphocytes enriched in the lower interface were collected and washed, respectively. To establish Pa and Ra, some tumor cells from primary and recurrent tumors were respectively cultured in RPMI1640 (Invitrogen, Grand Island, NY, USA), which was initially supplemented with 20% autologous serum and subsequently supplemented with 10% fetal bovine serum, once the autologous serum was depleted. The remaining tumor cells were continuously cultured in RPMI1640 (Invitrogen, Grand Island, NY) supplemented with 10% fetal bovine serum to obtain Pb cells from the primary tumor and Rb cells from the recurrent tumor. The cell cultures were regularly passaged via trypsinization. Notably, the cells used for analysis in the present study were passaged between 5 and 15 times (prior to the establishment of immortal cell lines) to obtain sufficient cell numbers and avoid bias resulting from long-term culture. TILs from recurrent tumors were cultured in RPMI-1640 medium supplemented with 2 mmol/l glutamine, 5.5 × 10^-5^ mol/l β-mercaptoethanol, 10% human AB serum (Blood Products Institute, Tianjing, China) and 6000 U/ml interleukin (IL)-2 (Ludesheng, Beijing, China).

### Flow cytometric DNA ploidy analysis

Pa, Pb, Ra, and Rb cells were subjected to flow cytometric DNA ploidy analysis, and human peripheral blood mononuclear cells (PBMCs) were used as normal cell control. Trypsin-digested single cells were centrifuged, washed with phosphate-buffered saline, fixed with 70% cold ethanol, and stained with DNA-specific fluorochrome propidium iodide (Sigma-Aldrich, St. Louis, MO) at a working concentration of 10 μg/ml. The fluorescence of propidium iodide was determined with a flow cytometer (BD FACSCalibur, BD Biosciences, Franklin Lakes, NJ).

### WGS

Genomic DNA of Pa, Ra, Pb, Rb, and TIL was extracted from the samples using the QIAamp DNA Mini Kit (Qiagen, Hilden, Germany) and used for WGS analysis. Libraries of Pa, Ra, and TIL were constructed from 1–3 µg DNA of each sample with the Paired-End DNA Sample Prep Kit (Illumina, San Diego, CA) according to the manufacturer's instructions, and paired-end sequencing of 2 × 100 bp was performed on the Illumina HiSeq 2000 platform at the core facility of the Beijing Institute of Genomics, CAS. To obtain more data, sequencing libraries of Pa, Ra, Pb, Rb, and TIL samples were generated using NEB Next® Ultra DNA Library Prep Kit for Illumina® (NEB, Boston, MA) following manufacturer’s recommendations, and paired-end sequencing of 2 × 150 bp was performed on the Illumina HiSeq 4000 platform at Novogene (Beijing, China). The average sequencing depths of WGS obtained were > 60 ×.

### WEs

WES libraries were constructed from 1–3 µg of DNA of Pa, Ra, Pb, and Rb samples with the Paired-End DNA Sample Prep Kit (Illumina) according to the manufacturer's instructions, and were further captured using the Agilent SureSelect Target Enrichment System (Human All Exon V4 kit, Santa Clara, CA). Paired-end sequencing of 2 × 100 bp and 2 × 125 bp was performed on the Illumina HiSeq2000 platform at the core facility of the Beijing Institute of Genomics, CAS and the Illumina HiSeq 2500 platform at BerryGenomics (Beijing, China), respectively. The average sequencing depths of WES were > 100 ×.

### RNA-seq

Total RNA was extracted from Pa, Ra, Pb, and Rb samples using the TRIzol reagent (Invitrogen). For each sample, 10 μg of total RNA was subjected to mRNA purification and used for library construction using TruSeq® RNA Sample Preparation v2 (Illumina) according to the manufacturer’s instructions. We performed two biological replicates for each sample using two sequencing platforms. Paired-end 2 × 100 bp sequencing was performed on the Illumina HiSeq 2000 platform at the core facility of the Beijing Institute of Genomics, CAS, and 2 × 125 bp sequencing was performed on the Illumina HiSeq 2500 platform at BerryGenomics. High Pearson correlation coefficients (∼0.95) were observed between the biological replicates.

### WGBs

We performed WGBS for Pa, Ra, and Rb samples. Purified genomic DNA of each sample was mixed with 0.1% lambda DNA, and sonicated using a Covaris S220 instrument (Woburn, MA, USA). End-repair, dA-tailing, and ligation were performed using the NEBNext End Repair Module, dA-Tailing Module, and NEB T4 ligase, respectively. Subsequently, DNA fragments were ligated with methylated adaptors. Size selection was performed by gel extraction to obtain DNA fragments larger than 200 bp. The adaptor-ligated DNA was treated with sodium-bisulfite using the EZ DNA Methylation-Gold™ Kit (Zymo, Irvine, CA), and subsequently amplified using KAPA HiFi HotStart Uracil + ReadyMix PCR Kit (KAPA Biosystems, Roche, Wilmington, MA) with 10 cycles. Paired-end sequencing of 2 × 100 bp was performed on the Illumina HiSeq 2000 platform at the core facility of the Beijing Institute of Genomics, CAS.

### WES and WGS data processing

Paired-end WES and WGS data were aligned to the human reference sequence (UCSC hg19) using the Burrows-Wheeler Aligner (BWA) [64]. All sequenced and aligned reads were further processed using both the Picard-tools and the Genome Analysis Toolkit (GATK) [65], including de-duplication, base quality recalibration, and multiple-sequence realignment prior to mutation detection. Summary of the sequencing data for each sample is provided in Table S9.

### CNA detection

We used Sequenza software [66] to estimate CNAs, cellularity, and ploidy in Pa, Pb, Ra, and Rb samples. For each WGS tumor sample, TILs were used as the normal control. Regions with 0 copies of the B allele were defined as LOH regions.

### SNV identification

We employed MuTect [67], a Bayesian framework, for detecting somatic SNVs. For both WGS and WES data from Pa, Pb, Ra, and Rb samples, TILs were used as the normal control for SNV calling. For WES data, SNVs were called within the capture regions (Agilent SureSelect Human all exon V4). SNVs showing a mutation frequency of > 10% and variant reads (double-strand support) of > 5 in a tumor sample and none in the TIL sample (as patient matched normal) were maintained. All SNVs were annotated using Oncotator [68].

### SNV validation

We performed Sequenom genotyping to validate the detected somatic SNVs in WES data. Since the SNVs observed in all four samples were unambiguous, we randomly selected 45 shared SNVs for genotyping in Pa, Pb, Ra, Rb, and TIL samples. In addition, all of the polymorphic SNVs observed in 1–3 cell culture samples were subjected to genotyping in all the five samples. Genomic positions for all validated SNVs were retrieved using hg19 as reference. The detailed procedures of primer design, multiplexed PCR and allele-specific extension, as well as variant allele frequency (VAF) calculation of Sequenom genotyping were performed according to Ling and colleagues [31].

### Construction of phylogenetic trees

We constructed a phylogenetic tree of the cell cultures using the Wagner parsimony method in the PHYLIP package [69]. After excluding SNVs located in LOH regions of the four samples, the remaining SNVs were used to construct the phylogenetic tree. Both lists of the SNVs called from WGS and WES data were used for tree construction.

### RNA-seq data analysis

**Supplementary Figure S7:**
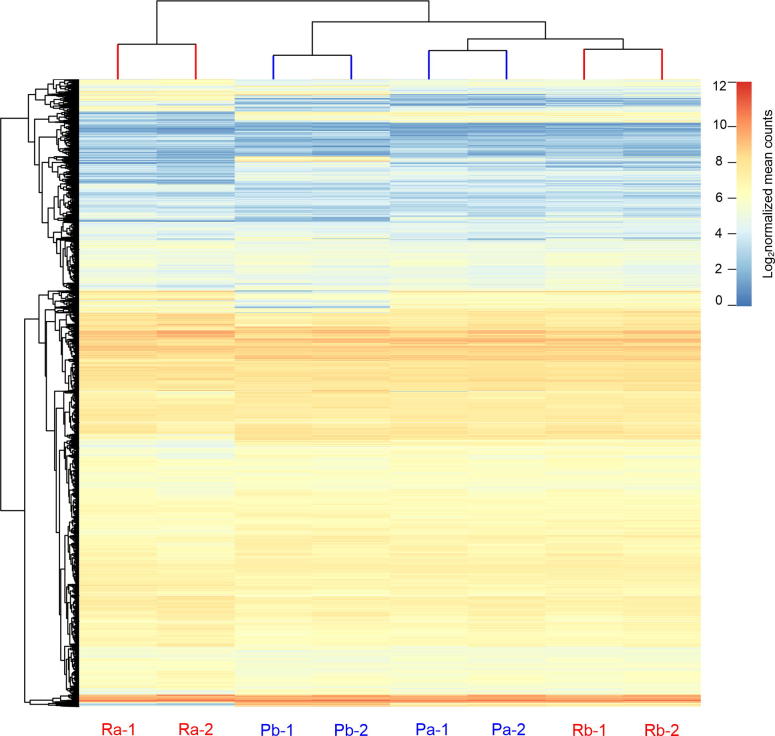
**Divergence of transcriptional profiles of biological replicates for each cell culture** Hierarchical clustering of biological replicates was performed based on normalized read counts on the log2 scale for genes that were expressed (read count > 1) in at least one sample. The heatmap depicts the expression of genes on the log2 scale (rows) from each replicate (columns).

Raw RNA-seq reads were aligned to the human reference sequence (UCSC hg19) using MapSplice [70]. Data summary is shown in Table S10. The aligned reads were sorted and indexed using SAMtools [71], and then translated to transcriptome coordinates. Indels, large inserts, and reads with zero mapping quality were filtered with UNC Bioinformatics utilities (UBU). We employed RSEM [72], an expectation maximization algorithm, to estimate the abundance of transcripts, which were subsequently annotated using information in Generic Annotation File 2.1 (GAF2.1). Raw RSEM expected counts for all samples were normalized to the overall upper quartile [73]. Subsequently, we employed log_2_-transformed values from the normalized counts for calculating PCCs between biological replicates, and for hierarchical clustering of replicates. The clustering pattern for the replicates was shown in Figure S7.

EBSeq [74], an empirical Bayesian approach, was used to identify DEGs from raw RSEM expected counts. For pair-wise comparison, we selected genes with a posterior probability of being differentially expressed > 0.99 and a fold change > 1.5 across two samples as DEGs. The specific DEGs of each sample were defined as the intersection of three lists of DEGs from comparisons between a specified sample and the other three samples.

**Supplementary Figure S8:**
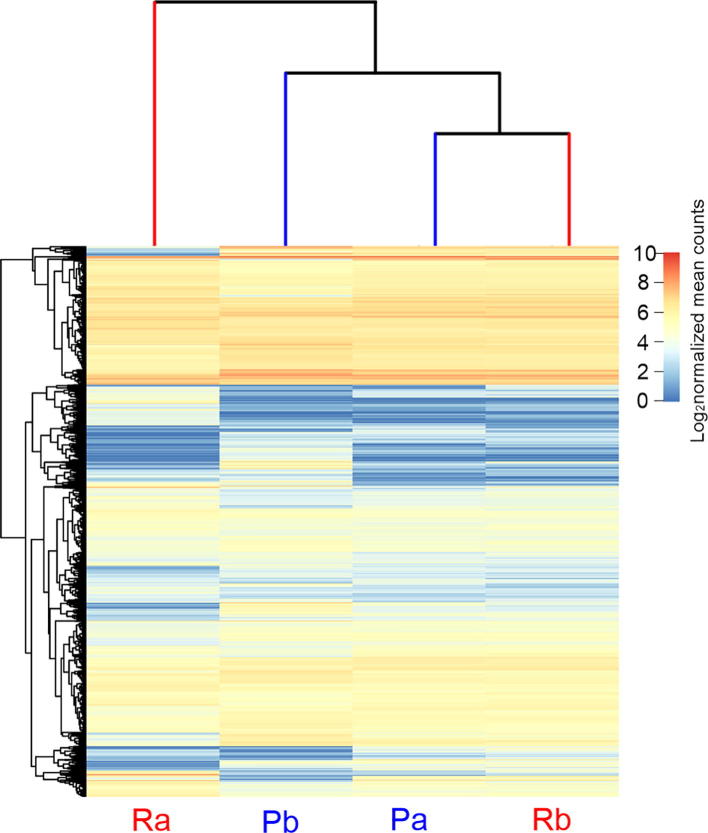
**Divergence of transcriptional profiles of the cell cultures based on DEGs** Hierarchical clustering of the cell cultures was performed based on normalized mean read counts on the log2 scale for DEGs between any two samples. The heatmap depicts the expression of DEGs on the log2 scale (rows) from each sample (columns).

The normalized mean counts of each gene (expressed in at least one sample) in each sample were log_2_-transformed values and employed for hierarchical clustering of samples using the Pheatmap package in R. Furthermore, DEGs were also employed for hierarchical clustering of samples. The clustering pattern based on DEGs was identical with the pattern based on all expressed genes (Figure S8).

### Cumulative distribution of gene expression across CNA regions

Empirical cumulative distribution of the expression of genes located in either gain or loss regions was illustrated for comparisons between any two of the four samples: Pa *vs.* Pb, Ra *vs.* Pa, Ra *vs.* Pb, Ra *vs.* Rb, Rb *vs.* Pa, and Rb *vs.* Pb. The gene expression was profiled based on normalized mean counts on the log_2_ scale and plotted using the R package. We employed the Kolmogorov–Smirnov test to calibrate the influence of CNAs on gene expression. The corresponding *P* values were calculated using the one-sided Kolmogorov–Smirnov test. Genes that were not expressed or showed low expression (count <  1) in the compared samples were ruled out in this analysis.

### WGBS data processing

Raw WGBS sequence reads were trimmed to remove low-quality bases, adaptor contamination, and poor-quality reads using Trimmomatic with default parameters [75]. Sequence reads of < 40  bp were excluded from further analysis. Trimmed sequences were aligned to the human reference sequence (UCSC hg19) using Bismark [76], and Picard-tools was used to remove duplications. Sequencing data summary is shown in Table S11. The methylation calls provided by Bismark were employed to extract the methylation statistics of CpG sites. Only CpG sites covered by > 10 reads in all samples were retained for further analysis. The methylation level of each CpG site was defined as the percentage of methylated counts.

### Annotation of functional genomic categories

Genome annotation tables were downloaded from UCSC Table Browser for functional genomic categories annotation [77], including CpG islands, transcripts, and repetitive elements in hg19. CpG island shores were defined as the 2 kb flanking regions of CpG islands, and CpG island shelves were defined as the 2 kb flanking regions of CpG island shores. For each RefSeq transcript, the promoters were defined as the regions spanning 1500 bp upstream to 500 bp downstream of transcription start site (TSS), and gene body regions were defined as the regions between TSSs and transcription end sites (TESs). 5′UTR, exons, introns, and 3′UTR were defined according to the RefSeq gene table. Several major repetitive element annotations including long interspersed nuclear element (LINE), short interspersed nuclear element (SINE), long terminal repeats (LTR), and satellite, as well as other repetitive DNA elements, were used from the RepeatMasker table. The methylation level of all functional genomic categories was calculated as the average methylation level of CpG sites within the regions containing these sites.

### DNA methylation analysis

To detect differentially methylated sites (DMSs) between each pair of samples, Fisher’s exact test was applied to the counts of methylated CpG site and total counts of CpG sites. CpG sites with a corrected *P* value (adjusted by Benjamini–Hochberg procedure) < 0.05 were identified as DMSs. Hierarchical clustering of methylomes was based on methylation levels of DMSs and was performed using the Pheatmap package in R. Furthermore, all the common CpG sites (> 10 ×) found in all the four samples were also employed for hierarchical clustering of samples. The clustering pattern based on all the common CpG sites was identical with the pattern based on DMSs.

We defined differentially methylated functional genomic categories (DMFs) as the categories showing differences in methylation levels of > 20% between any two samples (two-tailed Fisher’s exact test, *P* < 0.05). The methylation levels of all DMFs were calculated as the average methylation levels of CpG sites within the regions containing these sites. We used the methylation levels of DMFs for hierarchical clustering of functional categories.

### Plot distribution of DMPs/DMGBs with associated gene expression changes

The methylation levels of DMPs/DMGBs were considered to be the average methylation levels of CpG sites within these regions. For pair-wise comparisons, the methylation differences (ΔML) of DMPs/DMGBs were plotted with expression changes (fold change) of corresponding genes.

### Functional/pathway enrichment analysis

We performed functional enrichment for Ra-specific DEGs using DAVID [78]. All the CNA/DNA methylation-driven genes of Ra were subjected to pathway enrichment analysis to obtain the significant enriched KEGG pathways via DAVID [78]. Subsequently, we employed CNA-driven/DNA methylation-driven genes and other Ra-specific DEGs participating in these pathways to build the network via Cytoscape [79].

### Cell morphology characterization

The cells were plated onto poly-l-ornithine (Sigma)-coated glass coverslips. After overnight culture, the cells were fixed, permeabilized and stained with Alexa Fluor® 488 Phalloidin (Molecular Probes) at room temperature for 20 min and subsequently washed. Finally, the cells were counterstained with DAPI. The slides were analyzed using an Olympus FluoView™ FV1000 Confocal Microscope.

### Anchorage-independent growth assay

Cells were suspended in 0.3% agar/ RPMI 1640 and plated at a density of 5000 cells per well onto 6-well plates that were previously coated with 0.5% agar. A 200 μL aliquot of fresh medium was added to each well every 3 days. After 3 weeks, the colonies were quantified without magnification, and the images were subsequently recorded using a stereomicroscope (Olympus, Tokyo, Japan).

### Tumorigenicity assay in NOD/SCID mice

For the tumorigenicity assay, 5 × 10^6^ cells were suspended in 100 μl of physiological saline and transplanted subcutaneously into the armpits of 4- to 6-week-old NOD/SCID male and female mice (Vital River Laboratories, Beijing, China). Tumor formation was monitored weekly. All the animal experiments were performed under a protocol approved by Peking University Cancer Hospital Animal Care and Use Committee.

## Data availability

The sequence data reported in this paper have been deposited in the Genome Sequence Archive [80] at the BIG Data Center, Beijing Institute of Genomics (BIG), CAS (GSA:CRA000636) that are publicly accessible at http://bigd.big.ac.cn/gsa.

## Authors' contributions

XL and XX designed, supervised, and coordinated the study. SL performed the study, analyzed and interpreted the data. ZY, CL, XL, and XX interpreted data. YL, QG, and XW was involved in processing the sequencing data. GL was involved in analyzing the sequencing data. XX, ZZ, and BX coordinated patient recruitment and established the primary cultures. TL was involved in data management and experimental design. SL wrote the manuscript with the help of ZY and CL. XL and XX revised the manuscript. All authors read and approved the final manuscript.

## Competing interests

The authors have declared no competing interests.
