# Supplementary material for: Multi-omics Analysis of Primary Cell Culture Models Reveals Genetic and Epigenetic Basis of Intratumoral Phenotypic Diversity
Source: Genomics Proteomics Bioinformatics. 2020 Mar 20;17(6):576–89. doi: 10.1016/j.gpb.2018.07.008 (PMC7212478; doi:10.1016/j.gpb.2018.07.008)
Supplement: Supplementary Table S5 [file mmc5.docx]

| **Table S5 Methylation level of the functional genomic categories** | | | | | | |
| --- | --- | --- | --- | --- | --- | --- |
| **Category** | **Mean (Pa)** | **Mean (Ra)** | **Mean (Rb)** | ***P* value (Pa and Ra)** | ***P* value (Pa and Rb)** | ***P* value (Ra and Rb)** |
| Promoter | 0.2358 | 0.1560 | 0.1904 | *P* < 2.2E−16 | *P* < 2.2E−16 | *P* < 2.2E−16 |
| 5'UTR | 0.5065 | 0.4515 | 0.4621 | *P* < 2.2E−16 | *P* < 2.2E−16 | *P* < 2.2E−16 |
| 3'UTR | 0.5615 | 0.4915 | 0.5112 | *P* < 2.2E−16 | *P* < 2.2E−16 | *P* < 2.2E−16 |
| Exon | 0.5344 | 0.4532 | 0.4884 | *P* < 2.2E−16 | *P* < 2.2E−16 | *P* < 2.2E−16 |
| Intron | 0.5768 | 0.5276 | 0.5348 | *P* < 2.2E−16 | *P* < 2.2E−16 | *P* < 2.2E−16 |
| Intergenic | 0.3356 | 0.3055 | 0.2804 | *P* < 2.2E−16 | *P* < 2.2E−16 | *P* < 2.2E−16 |
| CGI | 0.1802 | 0.1202 | 0.1479 | *P* < 2.2E−16 | *P* < 2.2E−16 | *P* < 2.2E−16 |
| CGIshore | 0.4574 | 0.3240 | 0.3795 | *P* < 2.2E−16 | *P* < 2.2E−16 | *P* < 2.2E−16 |
| CGIshelf | 0.5632 | 0.4526 | 0.4990 | *P* < 2.2E−16 | *P* < 2.2E−16 | *P* < 2.2E−16 |
| Other repetitive DNA elements | 0.4579 | 0.4230 | 0.4074 | *P* < 2.2E−16 | *P* < 2.2E−16 | *P* < 2.2E−16 |
| LINE | 0.3716 | 0.3542 | 0.3295 | *P* < 2.2E−16 | *P* < 2.2E−16 | *P* < 2.2E−16 |
| LTR | 0.3800 | 0.3337 | 0.3223 | *P* < 2.2E−16 | *P* < 2.2E−16 | *P* < 2.2E−16 |
| SINE | 0.5951 | 0.5223 | 0.5294 | *P* < 2.2E−16 | *P* < 2.2E−16 | *P* < 2.2E−16 |
| Satellite | 0.2267 | 0.0984 | 0.1547 | *P* < 2.2E−16 | *P* < 2.2E−16 | *P* < 2.2E−16 |
