# Supplementary material for: Multi-omics Analysis of Primary Cell Culture Models Reveals Genetic and Epigenetic Basis of Intratumoral Phenotypic Diversity
Source: Genomics Proteomics Bioinformatics. 2020 Mar 20;17(6):576–89. doi: 10.1016/j.gpb.2018.07.008 (PMC7212478; doi:10.1016/j.gpb.2018.07.008)
Supplement: Supplementary Table S8 [file mmc8.docx]

| **Table S8 Significantly enriched KEGG pathways of CNA/DNA methylation-driven genes** | | |
| --- | --- | --- |
| **ID** | **Pathway** | **Adjusted *P* values (Benjamini–Hochberg)** |
| hsa05200 | Pathways in cancer | 7.783E−04 |
| hsa04010 | MAPK signaling pathway | 4.454E−03 |
| hsa04151 | PI3K-Akt signaling pathway | 4.911E−03 |
| hsa04510 | Focal adhesion | 5.410E−03 |
| hsa04014 | Ras signaling pathway | 5.853E−03 |
| hsa04015 | Rap1 signaling pathway | 1.324E−02 |
| hsa04810 | Regulation of actin cytoskeleton | 1.207E−02 |
| hsa04360 | Axon guidance | 1.843E−02 |
| hsa05410 | Hypertrophic cardiomyopathy (HCM) | 3.053E−02 |
