# Supplementary material for: Multi-omics Analysis of Primary Cell Culture Models Reveals Genetic and Epigenetic Basis of Intratumoral Phenotypic Diversity
Source: Genomics Proteomics Bioinformatics. 2020 Mar 20;17(6):576–89. doi: 10.1016/j.gpb.2018.07.008 (PMC7212478; doi:10.1016/j.gpb.2018.07.008)
Supplement: Supplementary Table S9 [file mmc9.docx]

| **Table S9 WES and WGS data statistics** | | | | |
| --- | --- | --- | --- | --- |
| **Sample** | **No. of total reads** | **No. of mapped reads** | **Mapping rate** | **Effective average depth** |
| TIL.WGS | 1,669,120,456 | 1,540,859,800 | 0.923 | 60 |
| Pa.WGS | 1,712,484,233 | 1,566,591,185 | 0.915 | 60 |
| Pb.WGS | 1,317,151,304 | 1,313,814,218 | 0.998 | 62 |
| Ra.WGS | 1,775,899,709 | 1,649,153,732 | 0.929 | 62 |
| Rb.WGS | 1,413,628,304 | 1,409,378,633 | 0.997 | 66 |
| Pa.WES | 297,803,350 | 288,821,244 | 0.970 | 166 |
| Ra.WES | 398,789,072 | 387,624,816 | 0.972 | 201 |
| Pb.WES | 207,200,824 | 205,463,549 | 0.992 | 116 |
| Rb.WES | 269,779,235 | 266,143,819 | 0.987 | 151 |
