# Supplementary material for: Multi-omics Analysis of Primary Cell Culture Models Reveals Genetic and Epigenetic Basis of Intratumoral Phenotypic Diversity
Source: Genomics Proteomics Bioinformatics. 2020 Mar 20;17(6):576–89. doi: 10.1016/j.gpb.2018.07.008 (PMC7212478; doi:10.1016/j.gpb.2018.07.008)
Supplement: Supplementary Table S10 [file mmc10.docx]

| **Table S10 RNA-seq data statistics** | | | | |
| --- | --- | --- | --- | --- |
| **Sample** | **No. of total reads** | **Unique mapping rate** | **Multiple mapping rate** | **Proportion of unmapped reads** |
| Pa-1 | 91,457,248 | 0.9558 | 0.0229 | 0.0213 |
| Pb-1 | 97,002,332 | 0.9647 | 0.0187 | 0.0166 |
| Ra-1 | 96,722,130 | 0.9503 | 0.0274 | 0.0223 |
| Rb-1 | 96,042,876 | 0.9632 | 0.0218 | 0.015 |
| Pa-2 | 93,393,338 | 0.9914 | 0.0191 | 0.0074 |
| Pb-2 | 76,763,558 | 0.9799 | 0.0159 | 0.0042 |
| Ra-2 | 79,982,790 | 0.9696 | 0.0212 | 0.0092 |
| Rb-2 | 84,050,062 | 0.9736 | 0.0184 | 0.0079 |
