# Supplementary material for: Multi-omics Analysis of Primary Cell Culture Models Reveals Genetic and Epigenetic Basis of Intratumoral Phenotypic Diversity
Source: Genomics Proteomics Bioinformatics. 2020 Mar 20;17(6):576–89. doi: 10.1016/j.gpb.2018.07.008 (PMC7212478; doi:10.1016/j.gpb.2018.07.008)
Supplement: Supplementary Table S11 [file mmc11.docx]

| **Table S11 WGBS data statistics** | | | | | |
| --- | --- | --- | --- | --- | --- |
| **Sample** | **Sequence pairs** | **Mapping efficiency** | **Bisulfite conversion rate** | **C methylated in CpG context** | **Effective average depth** |
| Pa | 330,571,429 | 86.55% | 99.70% | 46.95% | 29.00 |
| Ra | 355,966,509 | 86.30% | 99.74% | 41.10% | 28.00 |
| Rb | 336,782,378 | 87.56% | 99.68% | 41.60% | 20.00 |
